# Molecular insights from the crab *Neohelice* memory model

**DOI:** 10.3389/fnmol.2023.1214061

**Published:** 2023-06-21

**Authors:** Arturo Romano, Ramiro Freudenthal, Mariana Feld

**Affiliations:** ^1^Universidad de Buenos Aires, Facultad de Ciencias Exactas y Naturales, Departamento de Fisiología, Biología Molecular y Celular “Dr. Hector Maldonado” (FBMC), Buenos Aires, Argentina; ^2^Biología Molecular y Neurociencias (IFIBYNE), Instituto de Fisiología, Universidad de Buenos Aires-CONICET, Buenos Aires, Argentina; ^3^Biotecnología y Biología Traslacional (IB3), Facultad de Ciencias Exactas y Naturales, Instituto de Biociencias, Universidad de Buenos Aires, Buenos Aires, Argentina

**Keywords:** memory, consolidation, reconsolidation, extinction, NF-kB, ERK, NMDAR, crab

## Abstract

Memory acquisition, formation and maintenance depend on synaptic post-translational machinery and regulation of gene expression triggered by several transduction pathways. In turns, these processes lead to stabilization of synaptic modifications in neurons in the activated circuits. In order to study the molecular mechanisms involved in acquisition and memory, we have taken advantage of the context-signal associative learning and, more recently, the place preference task, of the crab *Neohelice granulata*. In this model organism, we studied several molecular processes, including activation of extracellular signal-regulated kinase (ERK) and the nuclear factor kappa light chain enhancer of activated B cells (NF-κB) transcription factor, involvement of synaptic proteins such as NMDA receptors and neuroepigenetic regulation of gene expression. All these studies allowed description of key plasticity mechanisms involved in memory, including consolidation, reconsolidation and extinction. This article is aimed at review the most salient findings obtained over decades of research in this memory model.

## Introduction

The development of memory models in different species of invertebrates, such as mollusks and insects heightened the study of the molecular mechanisms involved in memory (e.g., Kandel, 2001; Menzel, 2001; Roberts and Glanzman, 2003; Crow, 2004; Margulies et al., 2005; Reissner et al., 2006; Giurfa and Sandoz, 2012; Hawkins and Byrne, 2015). In the last decades a research endeavor has been focused on the understanding of molecular mechanisms involved in learning and memory in a crustacean model organism, the crab *Neohelice granulata* (formerly named *Chasmagnathus granulatus*). This species has a great visual acuity and a particular sensitivity to the detection of moving objects above the visual horizon (Tomsic and Romano, 2013). This sensitivity is highly adaptive to detect and avoid natural predators, such as seagulls. Taking these features into consideration, our group, initially directed by Dr. Héctor Maldonado, developed in the 80s a laboratory task to study learning and memory. In this task, the presentation of an aversive stimulus, an opaque rectangular figure passing over the animal, initially elicits an escape response. The iteration of this danger stimulus (DS) provokes the decline of the escape response (Lozada et al., 1990). Thus, memory is revealed at testing by a significant decrease in response to the DS of a trained group of animals with respect to untrained animals in a control group. Long-term memory (LTM), lasting more than a week, is induced by a strong training of at least 15 spaced trials of stimulus presentation, using typically 171 s of inter-trial interval (ITI). Instead, massed training of 300 DS trials, with 4 s ITI, induced retention for no more than 3 days and a different mechanism than that of LTM is involved (Pedreira et al., 1998; Hermitte et al., 1999). Behavioral analysis of massed training suggests non-associative habituation process that is only present in the re-training phase of the testing session (after the first trial). Conversely, spaced training induced a context-dependent memory with associative characteristics (context-signal associative learning), showing traits of conditioning: latent inhibition and extinction (Tomsic et al., 1998). Furthermore, minimizing ITI to 0 s in a continuous stimulation training protocol, induced a short-term decrement of the response without long-term retention in spite of the huge increase of training trials used (e.g., 600 trials or more; Freudenthal et al., 1998). This kind of active control protocol provides a useful tool to reveal the effects of visual stimulation, stress, novelty, motor activity, etc., which could induce nonspecific molecular changes (e.g., not related to memory formation). The so-called Context-Signal Memory (CSM, named after the association between the stimulus and contextual cues) is evident mainly in the first testing trial but also in the retraining phase (Pedreira et al., 1998).

In order to make more salient the associative characteristics of this type of learning, modifications of the context presentation were introduced in order to be present simultaneously with the danger stimulus. Such modification was achieved by switching the illumination from below to above the device before and in coincidence with the stimulus presentation. This new learning task showed characteristics of contextual pavlovian conditioning (CPC; Fustiñana et al., 2013).

Tomsic and colleagues described movement detector neurons from the *lobula* of the third optic ganglion, using intracellular recordings *in vivo*. Notably, not only do these cells respond to the danger stimulus but they also adjust their activity during learning in close correlation with the behavioral characteristic of memory acquisition and retention. These results indicate that information of the danger stimulus during learning is processed at least in part within the optic neuropiles (Tomsic et al., 2003). Studying the supra-esophageal ganglion (central brain) we obtained an important body of molecular evidence regarding the formation and reprocessing of memory in this model (Romano et al., 2006).

The crab model proved to be very useful for the pharmacological approach to the study of memory, due to the high sensitivity and the rapid effect of systemic administration. This is easily achieved by the introduction of a syringe in the cephalothorax-abdominal membrane, allowing the introduction of a needle into the pericardial sac. Thus, the different drugs reach the heart and then are rapidly distributed to the central brain, among other organs, through the dorsal artery. With this method, the effect of several drugs was tested over the last decades in different phases of memory (Delorenzi et al., 1996; Berón de Astrada and Maldonado, 1999; Frenkel et al., 2002; Pedreira et al., 2002; Troncoso and Maldonado, 2002; Tomsic and Romano, 2013 for a review). The role of different neurotransmitters has been unveiled by means of these systemic intra-pericardial drug administrations.

In this review, we will focus on the molecular mechanisms carried out in this model organism, showing the characteristics of the different phases of memory.

## Memory consolidation

**Figure 1:**
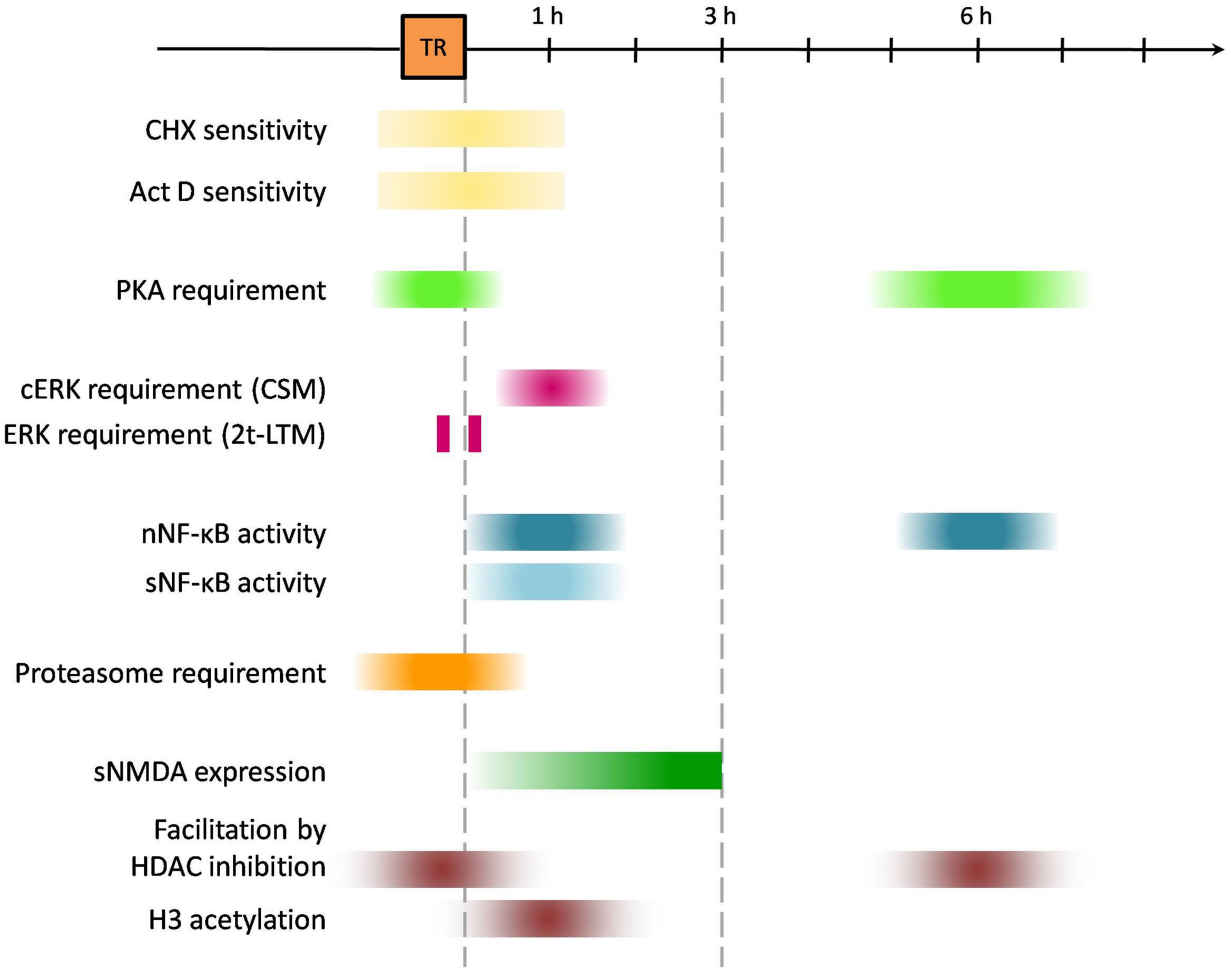
Schematic timeline for molecular pathways’ involvement in learning and consolidation using *Neohelice granulata* memory model. “TR” orange box indicates time for learning protocol (45 min approx); colored boxes suggest times relative to training at which either molecular pathways were found to be active or pharmacological interventions exerted any effect on memory processes. CHX, cycloheximide; Act D, actinomycin D; cERK, cytosolic ERK (extracellular signal-regulated kinases 1/2); nNF-κB, nuclear NF-κB (Nuclear factor kappa-light-chain-enhancer of activated B cells); sNF-κB, synaptic NF-κB; sNMDAR, synaptic N-methyl-D-aspartate receptor.

Dependency on macromolecular synthesis during and immediately after learning experience constitutes a universal feature of long-lasting forms of memory (Squire and Davis, 1981). Accordingly, only during the first hours after training proved CSM in *Neohelice* to be sensitive to protein and mRNA synthesis inhibition using either cycloheximide or Actinomycin D (Figure 1; Pedreira et al., 1995, 1996). Similarly, CPC consolidation was impaired by the administration of cycloheximide during memory consolidation (Fustiñana et al., 2013).

Fasted crabs habituated to a circular arena can be reinforced with a food pellet in a striped quadrant (SQ) of the otherwise plain white arena. These experimental animals expend more time in SQ than in the habituation session when evaluated 24 h after training. Moreover, they also spend more time in SQ than animals not reinforced during the first encounter with the arena (Control animals). This appetitive long-term conditioned place preference (CPP) of *Neohelice* has proven to be dependent on protein synthesis, as the 24 h retention is affected when the animals are injected with cycloheximide immediately after training (Klappenbach et al., 2021). Protein synthesis blockage during consolidation has shown to be disruptive for long-term memory retention of both aversive and appetitive memories in *Neohelice*, despite the sensorial input differences of both types of tasks.

### Role of PKA in memory consolidation

The first signal transduction mechanism studied was the cAMP-dependent signaling. An important body of evidence had implicated this pathway in neural plasticity (e.g., Klein and Kandel, 1978; Castellucci et al., 1982; Klein et al., 1986; Frey et al., 1993). We obtained data showing that the manipulation of PKA activity by means of the systemic administration of cAMP analogs affects memory formation.

Initially, we found that long-term memory can be facilitated by the administration of CPT-cAMP (a PKA activator) plus IBMX (a phosphodiesterase inhibitor) before or after a weak training (Romano et al., 1996a). Consequently, we concluded that high cAMP levels and PKA activation are important hallmarks in memory. However, use of these drugs did not discard involvement of the cGMP-PKG pathway. Therefore, to rule out this possibility, we used the membrane permeable cAMP analogues, Sp-5,6-DCl-cBIMPS and Rp-8-Cl-cAMPS, which are highly specific PKA activator or inhibitor, respectively. The PKA activator showed memory enhancement, whereas the PKA inhibitor induced memory impairment (Romano et al., 1996b).

Further analysis, using Rp-8-Cl-cAMPS, revealed two time points in which the drug is amnesic, indicating that CSM consolidation requires PKA activity in two critical periods: one during training and other between 4 and 8 h after training (Locatelli et al., 2002).

Next, to evaluate if PKA activation is in fact part of the cellular mechanisms triggered by training, we measured PKA activity in the central brain (supraesophageal ganglion) during the critical periods for CSM formation. In agreement with our previous findings, PKA was in fact activated in the brain immediately and 6 h after training (Locatelli and Romano, 2005). Further, we demonstrated that PKA I and PKA II isoforms are present in *Neohelice* neural tissues, showing characteristics similar to those described in other taxa. Indeed, PKA I showed 10-fold higher sensitivity than PKA II to cAMP. We found PKA II activation after exposure to the training context without stimulus presentation. In the case of animals trained in the CSM, we found a significant increase in the total level of PKA I 6 h after training. Considering the higher sensitivity of PKA I to cAMP, its increase can account for the PKA activation found 6 h after training and is proposed as a novel mechanism providing the prolonged PKA activation during memory consolidation (Locatelli et al., 2001).

### Mitogen activated protein kinases in CSM and CPC

Focusing on signal transduction mechanisms, the activities of mitogen activated protein kinase (MAPK) cascades have also been found to regulate synaptic plasticity (English and Sweatt, 1996; Martin et al., 1997; Michael et al., 1998; Winder et al., 1999; Guan et al., 2003). Some reports also described its role in relation to memory processes in rodents (Atkins et al., 1998; Berman et al., 1998; Blum et al., 1999; Walz et al., 1999; Schafe et al., 2000; Zhen et al., 2001; Alonso et al., 2002; Bevilaqua et al., 2003) as well as in invertebrates such as *Drosophila* (Pagani et al., 2009), *Hermissenda* (Crow et al., 1998), and *Aplysia* (Purcell et al., 2003; Sharma et al., 2003). MAPKs are a large family of Serine/Threonine protein kinases described in almost every tissue, regulating processes such as development, mitogenesis and the stress response, and play important roles in transducing external signals to maintain cellular homeostasis. These pathways typically consist of a 3-associated kinase motif in terms of their sequential activation (cascades) by phosphorylations. Thus, a MAPK kinase kinase phosphorylates and activates a MAPK kinase, which in turn phosphorylates and activates the so-called effector: the MAPK. Amplitude and duration of the signal is tightly regulated by positive and negative feedback mechanisms controlling phosphorylation of their activation loop. Moreover, MAPK pathways are further modulated by interactions of MAPKs with scaffold and regulatory proteins which also determine subcellular localization of the kinases activity and consequently the set of targets affected.

Two of the most studied MAPKs cascades are the highly conserved extracellular signal-regulated kinase (ERK) 1/2 and c-Jun N-terminal kinase (JNK) 1/2. They are present in a variety of species such as yeast, worms, mollusks, insects and mammals (Galcheva-Gargova et al., 1994; Noselli and Agnès, 1999; Villanueva et al., 2001). We have characterized the presence of both these kinases in their active, phosphorylated forms in the central brain (supraesophageal ganglion) of *Neohelice* (Feld et al., 2005). As it was described in other invertebrates (Buscà et al., 2016), we detected only one band of 43 kDa using a monoclonal antibody raised against human phospho-ERK in both cytosolic and nuclear protein extracts from central brains of *Neohelice* (Feld et al., 2005). Confocal microscopy analysis of the central brain also showed pERK immunoreactivity mainly in the olfactory lobes, in the olfactory globular tract and through the protocerebral tract. Regarding JNK, using a monoclonal human phospho-specific antibody, we were able to detect two bands of similar molecular weights to those of mammal JNK1 (p46) and JNK2 (p54). They also were detected in central brain nuclear as well as extranuclear protein extracts. Visualization of crab central brain confocal images confirmed pJNK1/2 presence mainly in nerves and glomeruli from the olfactory lobe, in some nuclei of the VI group somas and in particular layers of the medulla in the optic ganglia.

In terms of memory processes, we initially studied CSM-induced ERK and JNK1/2 activation in central brains of *Neohelice* and observed a differential regulation of these protein kinases in extra-nuclear protein-enriched homogenates (Feld et al., 2005): while spaced training induced ERK specific activation 1 h later, both pERK and pJNK1/2 peaked immediately after passive and active controls (e.g., no stimulation or continuous stimulation, respectively). Although nuclear activation was not revealed, these findings suggested a specific role for ERK in associative memory consolidation. Therefore, we hypothesized that ERK inhibition would result in memory impairment. Thus, we used PD098059 (PD) systemic administration, which inhibited *Neohelice’s* ERK activation in a dose-dependent manner, in order to test this prediction. Infusion of 20 ug of PD (inhibiting 40% activation approximately) either immediate pre- or post-training did not affect LTM 48 h after training. However, specific administration of PD 45 min after training (15 min prior to CSM-induced peak of ERK activity) induced LTM impairment (Feld et al., 2005) without affecting STM (Feld, unpublished results). Moreover, PD effect on LTM was also dose-dependent as 30 ug, but not 10 ug, abolished retention. These results confirmed cytosolic ERK involvement in memory consolidation in the CSM of the crab *Neohelice granulata*.

At variance with consolidation, reconsolidation did not depend on either ERK or JNK activity since we found no phospho-ERK or phospho-JNK changes up to 3 h after CSM reactivation.

We recently found that using CPC, it is possible to induce LTM (tested 24 up to 96 h after training) when only 2 trials are delivered, as long as they are spaced by 45 min (2 t-LTM hereafter). Neither 3 nor 60 min intertrial intervals allowed LTM retrieval (Ojea Ramos et al., 2021). This finding was consistent with previous findings in mollusks (Philips et al., 2007; Liu et al., 2017). This observation constitutes an extreme case of spacing effect, a phenomena described during learning in different experimental memory models, encompassing both invertebrates (Philips et al., 2007; Pagani et al., 2009; Ojea Ramos et al., 2021), as well as vertebrates (Ebbinghaus, 1885; Vlach et al., 2008; Bello-Medina et al., 2013; Aziz et al., 2014; Pandey et al., 2015). We characterized 2 t-LTM in terms of protein synthesis dependency of consolidation, reconsolidation and context specificity finding that it resembles other kinds of associative memories. Moreover, ERK inhibition immediately pre- or post-training also abolished long term retention. Strikingly, we observed an increase in ERK activity 30 min after the first trial, but 5 min after the second one, suggesting that a second trial after 45 min ITI accelerates the activation peak. Noteworthy, PD systemic administration, either 15 or 22 min (but not immediately or 30 min) after the first trial, abolished 2 t-LTM. These results are in line with the ones using CSM since PD administration needs to be performed some minutes prior to ERK peak activity in order to effectively impair LTM. Importantly, they establish ERK as a relevant signaling pathway for LTM processing in the crab *Neohelice granulata*.

ERK activity in the crab’s nervous system has also been shown to be relevant in memory impairment induced by beta-amyloid, a peptide originated by cleavage of amyloid precursor protein (APP) which is a major constituent of the neuritic plaques found in Alzheimer’s disease patients and is proposed to be the cause of memory deficit and neurodegeneration observed in this pathology. Using CSM, we observed that very low doses of aggregated beta-amyloid compounds induced amnesic effects only when administered immediately pre- and post-training, but not 24 and 18 h before or 6 h after training. Activation of nuclear and cytosolic ERK was found 60 min, but not 15 min, after administration. In contrast, phospho-JNK1/2 levels were not altered in this species, suggesting a specific effect on CSM-induced signaling which accounted for memory impairment (Feld et al., 2008). These findings highlight that invertebrate memory models might be useful tools to study neuropathological disorders.

### Participation of Rel/NF-kB transcription factor in memory consolidation

Increasing experimental data supports the regulation of gene expression in LTM formation. Nuclear transcription factor κB (NF-κB) is highly expressed in the nervous system. In neurons it is localized in the soma but also in neurites and particularly in synaptic terminals (Kaltschmidt et al., 1993; Meberg et al., 1996; Suzuki et al., 1997). This striking localization led to the question of why a transcription factor is present in synapses and so far from its canonical role in the nucleus. The first evidence indicating that learning activates NF-κB was found by our group in the crab *Neohelice* memory (Freudenthal et al., 1998; Freudenthal and Romano, 2000). Due to the lack of genetic information of NF-κB family members in this species, we took advantage of the highly conserved nucleotide consensus sequences or elements specifically recognized by transcription factors. By means of the use of gel shift assay, we were able to detect specific κB DNA binding activity using nuclear protein extracts from the crab’s brain. A double strand DNA oligonucleotide containing the NF-κB consensus sequence was used to incubate with these extracts. Using gel shift electrophoresis, we detected a specific complex in the crab central brain. One of the complex components is a 61 kD protein, identified with an antibody against p65/Rel A, a member of NF-κB family in mammals. With this technique we obtained an estimation of nuclear NF-κB activity in the crab central brain which highly correlated with CSM formation observed after a spaced training. On the contrary, NF-κB was not activated after massed training. Two phases of NF-κB activation were found after spaced training of 10 or more trials, which induce LTM formation: the first one was peaking immediately after training and decaying to basal level 3 h afterwards and the second one increasing 6 h after training and waning to basal level 12 h after training (Freudenthal and Romano, 2000). Strikingly, activation of NF-κB was found after spaced training in synaptic terminals. This finding supports the hypothesis of the dual role of NF-κB in synapse-to-nucleus signaling mechanisms, initially as a synaptic activity detector and then as a transcription factor after its translocation to the nucleus (Meberg et al., 1996; Freudenthal and Romano, 2000; Wellmann et al., 2001; Meffert et al., 2003).

After the strong correlation found in these previous reports between NF-κB activation and the training conditions for LTM formation, we next evaluated the necessity of this pathway of gene expression by means of the drug sulfasalazine. This drug specifically inhibits the protein kinase that activates NF-κB, IκB kinase (IKK). Sulfasalazine administration induced amnesia at the same time NF-κB was found to be active but not before or after the activation phases (Merlo et al., 2002). Given the similitude between NF-κB and PKA activity profiles after training, an interrogant that remains elusive is whether these two pathways might be related in memory formation.

The protein kinase IKK induces the activation of NF-κB by phosphorylation of the IκB inhibitor protein. The phosphorylation signals IκB for ubiquitination and posterior degradation by the ubiquitin-proteasome system (UPS; Ghosh et al., 1998). Thus, inhibiting UPS might have an effect on memory formation by inhibiting NF-κB activation. A cell-permeable 26S proteasome inhibitor, MG132, was used to test this hypothesis. MG132 impaired training-induced NF-κB activation in the crab brain when administered *in vivo*. In turn, the same dose also impaired LTM (Merlo and Romano, 2007). Similar result was obtained using the CPC task of the crab (Fustiñana et al., 2013).

Another question that we devised was what neurotransmitters or neuromodulators trigger NF-κB activation during learning. The neuropeptide angiotensin II plays a key role in the crab LTM consolidation and activates nuclear NF-κB in the central brain at the same doses that also facilitate CSM. The activation of the transcription factor was reversed by the angiotensin II receptor antagonist Saralasin. Furthermore, hydric stress also induced NF-κB activation, increasing at the same time angiotensin II in the brain and facilitated LTM expression (Frenkel et al., 2002).

### Surface expression of NMDA like receptor dynamics during long-term memory consolidation

The N-methyl-D-aspartate receptor is known to be a coincidence detector required at the synapse for a variety of vertebrate and invertebrate learning paradigms (Rezvani, 2006; Glanzman, 2009), critical for controlling synaptic plasticity and memory formation (Morris, 2013), as is the case for CSM consolidation and reconsolidation in *Neohelice granulata* (Pedreira et al., 2002; Troncoso and Maldonado, 2002).

A NMDA receptor-like (NMDAR) protein was recognized in the nervous system of *Neohelice granulata* using antibodies directed to the carboxyl-terminus of the GluN1 subunit of the rodent receptor (Hepp et al., 2013). Immediately or 3 h after a 30-trial training of CSM (strong training) the overall expression of the GluN1 subunit in the central brain of the crab did not change compared to control and naive animals. Nonetheless, the ratio between synaptic membrane and intracellular pool GluN1 (NMDAR surface expression) in the central brain changed significantly compared to control and naïve animals. Immediately after training, trained animals showed a significant surface expression decrease in comparison with control and naïve animals, whereas 3 h post training, the same group showed a significant increase compared to naïve animals, and a tendency to increase compared with control animals. Twenty four hours post training, the surface expression of NMDAR in the central brain showed no significant differences among groups. These changes in proportion of membrane NMDAR occur only in the central brain and not in the thoracic ganglion, a structure of the nervous system associated with locomotion, adding evidence to the role of the central brain in CSM long-term memory consolidation (Hepp et al., 2016).

We hypothesize that these changes in the GluN1 surface expression of trained animals during consolidation constitute a form of metaplasticity. Thus, changes in membrane NMDAR might underlie a shift in the threshold to undergo further plasticity. Following this hypothesis, the synapses involved in this task will be less likely to be involved in NMDA related changes immediately after training, and more likely involved in this type of plasticity 3 h post training. These results point to a putative memory maturation system involving a regulation of the probability of adding further information to the trace.

### Histone acetylation as a neuroepigenetic mechanism in memory formation

Using the CSM task our group has focused a line of research on histone acetylation during memory consolidation and its relation with memory strength. For this purpose, we trained the animals with 15 trials of standard protocol or 30 trials of strong training. We studied the levels of histone H3 acetylation in *Neohelice* central brain during consolidation and we found an increase in that levels only after the strong training (Federman et al., 2009). We also found that the memory induced by strong training was resistant to extinction. The resistance to extinction is considered as indicative of memory strength (Tully and Quinn, 1985; De Oliveira Alvares et al., 2013). In contrast, standard training memory was affected by extinction treatment (Federman et al., 2012). Furthermore, we trained crabs with a weak training protocol of 5 trials and we administered inhibitors of histone deacetylases (HDACs), sodium butyrate or Trichostatin A during memory consolidation. Both inhibitors induced memory enhancement (Federman et al., 2009). Although the first evidence for the role of this epigenetic mechanism in neuronal plasticity was obtained in *Aplysia* (Guan et al., 2002), our work in *Neohelice* constituted the first direct evidence of the role of chromatin modifications during the formation of the LTM in invertebrates.

**Figure 2:**
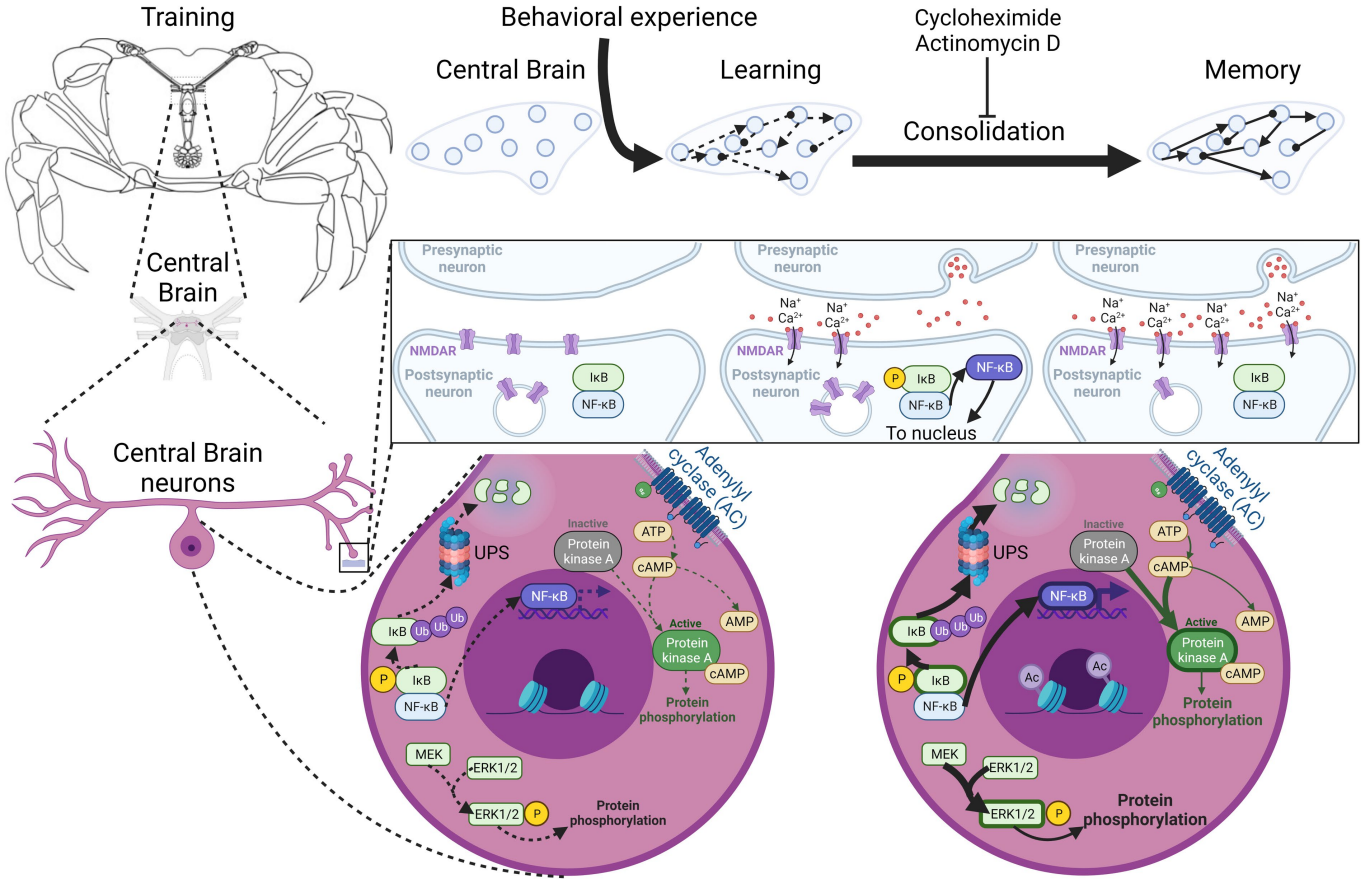
Localization of molecular pathways that participate in *Neohelice granulata* long-term memory consolidation. Crab central nervous system disposition and ganglia studied are shown in the left. Subcellular positioning of the pathways identified are correlated with memory processes depicted at the top of the figure, highlighting subcellular localization of main pathways discussed in the text. Schematically, all these pathways are represented in only one neuron, although different types of neurons involved in different pathways could be present in the central brain. NMDAR, N-methyl-D-aspartate receptor; NF-κB, Nuclear factor kappa-light-chain-enhancer of activated B cells; IκB, nuclear factor of kappa light polypeptide gene enhancer in B-cells inhibitor; UPS, ubiquitin-proteasome system; ERK1/2, extracellular signal-regulated kinases 1/2; MEK, mitogen-activated protein kinase/ERK kinase; P, phosphorylation; Ub, ubiquitination; Ac, acetylation. Created with BioRender.com.

In Figure 1, the timeline of the different mechanisms activated by training is depicted as described in this memory consolidation section. In Figure 2, the localization of these processes is shown.

## Memory reconsolidation

Memory is initially labile and becomes stable and resistant to different treatments during consolidation. However, other studies showed that once consolidated a memory may become labile again when retrieved and reactivated by a reminder (labilization), opening a new consolidation-like period called reconsolidation (Misanin et al., 1968; Nader et al., 2000; Sara, 2000). Influential work in the crab CSM revealed that reconsolidation is not restricted to mammals and found important characteristics and boundary conditions. In CSM reconsolidation studies, once memory is consolidated, a brief re-exposure of 5 min to the training context, in which memory is acquired, reactivates the memory. This remainder opens a new period of lability in which protein synthesis inhibitors are again amnesic. However, protein synthesis inhibition was not amnesic when the animals were exposed to a context different from that used in training (Pedreira et al., 2002).

### NF-κB and memory reconsolidation

Reconsolidation showed some mechanistic similarities and differences from the initial consolidation phase (Debiec et al., 2002; Lee et al., 2004). We tested if NF-κB is required for reconsolidation as it is for consolidation, and found that it was specifically reactivated in animals re-exposed to the same training context but not to a different context. Conversely, NF-κB was not activated in animals re-exposed to the context after a weak training that is unable to induce long-term memory. In addition, the administration of sulfasalazine, the NF-κB inhibitor, impaired reconsolidation only shortly before re-exposure to the training context. Even more, sulfasalazine was ineffective when a different context was used instead (Merlo et al., 2005). These findings reveal for the first time that NF-κB is activated specifically by retrieval of the consolidated association and that this activation is required for memory reconsolidation. These data support the key role of this molecular mechanism in both consolidation and reconsolidation.

### Protein degradation in memory destabilization

As mentioned in a previous section, the UPS inhibition during consolidation impairs CSM (Merlo and Romano, 2007) and CPC memory (Fustiñana et al., 2013). However, when the same inhibitor, MG132, is administered previous to the re-exposure session, no amnesic action in CPC was found. The amnesic effect is expected in this case because NF-κB is required in memory reconsolidation and, as previously mentioned, the UPS is necessary for the transcription factor activation. Consistently, inhibition of the UPS during re-exposure blocks the amnesic effect induced on reconsolidation by sulfasalazine. The last finding suggests a specific action of the UPS inhibitor on memory labilization. Thus, once the labilization or destabilization process is impeded, reconsolidation does not take place and NF-κB activation is no longer required (Fustiñana et al., 2013). We note that proteasome inhibition may also impair memory by blocking degradation of additional key proteins, such as other transcriptional repressors (Chain et al., 1999).

### Chromatin acetylation in reconsolidation

It is unclear whether histone acetylation is involved in reconsolidation. We found an increment of histone H3 acetylation 1 h post-reactivation of CSM induced by strong training, but not for a memory formed by weak training. Sodium butyrate, an inhibitor of class 1 HDACs enhances memory during consolidation but was unable to induce memory enhancement during reconsolidation. However, a weak memory that was enhanced during consolidation by sodium butyrate recruited H3 acetylation in reconsolidation. These results suggest a role of histone acetylation in the re-stabilization of memory (Federman et al., 2012).

## Memory extinction

As mentioned in the previous section, a brief re-exposure to training contextual cues elicits memory reconsolidation. Conversely, the prolonged re-exposure of the crabs to the training container for more than 1 h induced memory extinction. A day after the prolonged re-exposure, the animals show high levels of escape response. Such an increment in escape response is considered a process of memory extinction, in which the originally conditioned response (low level of escape) is diminished. As observed for consolidation of the original memory, extinction consolidation is sensitive to CHX when administered soon after re-exposure but not 6 h later (Pedreira and Maldonado, 2003). Thus, studies using CHX revealed that memory retrieval could lead to either reconsolidation or extinction, depending on the remainder duration. This was a key finding for the understanding of memory dynamics and processing. As mentioned in the previous section, NF-κB plays a critical role in consolidation as well as reconsolidation of memory. Therefore, we then investigated the role of NF-κB in memory extinction. Under the hypothesis that extinction involves a new memory consolidation, with similar mechanistic features as the original one, we would expect the requirement of NF-κB for extinction consolidation. However, the administration of the NF-κB inhibitor sulfasalazine prior to the extinction session enhanced extinction rather than impair it. Consistently, reinstatement experiments showed that the original memory was intact. This fact supports that sulfasalazine had no amnesic effects and that sulfasalazine NF-κB inhibition may be either impairing or delaying spontaneous recovery, rendering memory extinction stronger. In fact, after memory consolidation, 5 min re-exposure to the training context induced activation of the transcription factor in the central brain, whereas prolonged re-exposure exerted inhibition 45 min after its beginning, and restoration to basal levels at 2 h, when a clear extinction level was already acquired (Merlo and Romano, 2008). Together, the data on the NF-κB activity dynamics in the crab’s brain provides new information about the molecular mechanisms involved in the switch that triggers either memory reconsolidation or extinction depending on re-exposure length. Among these mechanisms, an active NF-κB inhibition during prolonged re-exposure might prompt extinction consolidation. Consequently, these findings support a working model for the role of NF-κB in memory after retrieval. Memory reactivation would induce transcriptional activation mediated by protein kinases. In particular, IKK and PKA activation induces NF-κB activity and nuclear translocation (Ghosh et al., 1998). The prolonged presence of the training context would induce activation of other mediators such as the protein phosphatase calcineurin, which may induce NF-κB nuclear exportation, as was found in rodents (de la Fuente et al., 2014), decreasing nuclear activity of this transcription factor. This proposal involves that during memory reactivation, the administration of NF-κB inhibitors would enhance the effect of prolonged exposure to the context, strengthening extinction. We conclude these results are in line with the view that extinction formation involves some mechanisms that may differ from the ones recruited by the original memory consolidation, and extinction might engage weakening of the original consolidated circuits. To summarize, the evidence revised here supports the view that inhibition of NF-κB would be a critical step for memory extinction, making a difference with consolidation and reconsolidation, which on the contrary require its activation.

### Memory extinction as model for information integration

As described in a previous section, NMDA-type receptors surface expression in animals trained with a strong CSM training protocol showed a significant decrement immediately after training, a significant increase 3 h after training and no differences 24 h post training when compared to Naive animals (Hepp et al., 2016).

NMDA-type receptors in *Neohelice granulata* are necessary for the acquisition but not for the consolidation of extinction memories (Pérez-Cuesta et al., 2007), and as CSM consolidation entails significant changes in the surface expression of NMDA-like receptor in the central brain of the crab, we decided to test the hypothesis that long-term CSM extinction would be impaired at the time points at which the NMDA-like receptor surface expression is altered following the original CSM training (Rosso and Freudenthal, 2021).

To test this hypothesis, we studied the memory extinction efficacy of a short and a long reexposure protocol at the following times: immediately, 3 and 24 h after the CSM training protocol. To evaluate extinction the testing sessions were 24 or 48 h after training when the time of reexposure required it.

The long protocol shows extinction learning occurs when the re-exposure is delayed 24 h from training, but fails when applied immediately after it. When the short extinction protocol is used, no extinction is seen when applied immediately after or 24 h after the training session. Only when the short reexposure protocol is applied 3 h after CSM training, extinction is detected at the 24 h testing.

These results suggest that the extinction acquisition is impaired when the surface expression of NMDA-type receptors is low, and facilitated when the surface expression levels are high compared to naive animals.

The fact that an increase in the surface expression of the NMDA-type receptor is observed at this time point suggests a correlation between the acquisition of the original memories and the surface expression of this receptor.

The surface expression dynamics of the NMDAR would be reflected in the probability of occurrence of a second learning event, with sensory information similar to the first one. This may indicate a maturation process of the first trace, in terms of its integration capacity with further information, in which the NMDAR plays an important role.

## Concluding remarks

More than three decades of intense research efforts rendered a description of important signaling pathways and molecular mechanisms involved in memory, some of them described for the first time in the literature using the *Neohelice* learning and memory model. Over these years, the use of this model proved to be very suitable for behavioral, pharmacological and molecular strategies in the study of the different phases of memory processing. The complementary approaches using different models of memory in different *taxa* is mandatory for the better understanding of such a complex phenomena, and the comparative study allows the characterization of key molecular mechanisms of memory processing that are conserved in evolution. In fact, some mechanisms initially described in *Neohelice* proved to be also important in other *taxa*, such as mammals. This comparative strategy with different animal models has paved the way for the understanding of key mechanisms underlying memory formation and maintenance, as well as memory impairments.

## Author contributions

AR, RF, and MF contributed to conception and writing of the manuscript. MF designed the Figure 1. RF designed the Figure 2. All authors contributed to manuscript revision, read, and approved the submitted version.

## Funding

This lines of research were financed by ANPCYT, CONICET, and University of Buenos Aires grants.

## Conflict of interest

The authors declare that the research was conducted in the absence of any commercial or financial relationships that could be construed as a potential conflict of interest.

## Publisher’s note

All claims expressed in this article are solely those of the authors and do not necessarily represent those of their affiliated organizations, or those of the publisher, the editors and the reviewers. Any product that may be evaluated in this article, or claim that may be made by its manufacturer, is not guaranteed or endorsed by the publisher.
